# A low-cost autonomous and scalable hydroponics system for space farming

**DOI:** 10.1016/j.ohx.2025.e00625

**Published:** 2025-01-18

**Authors:** Jae Hyeon Ryu, Jeonghyun Baek, Zarin Subah

**Affiliations:** a University of Idaho, Boise, ID 83702, USA; b National Institute of Agricultural Sciences, Rural Development Administration, Jeonju 54875, Republic of Korea

**Keywords:** Space farming, Hydroponics, Aquaponics, Low-cost, Controlled environment, Basil

## Abstract

An alternative food production system using hydroponics is proposed to grow vegetables in a controlled environment that is implementable in space. The proposed system is an autonomous, modular, scalable, and soilless food production platform (ASFP) that can be installed in a spacecraft by meeting requirements and constraints set by the National Aeronautics and Space Administration (NASA). A suite of Internet of Things (IoT) sensors was used to monitor indoor climate as well as water quality in ASFP. Average values of air temperature and relative humidity in the environmentally-controlled room are maintained between 20–24 °C and 48–62 %, while water quality components, including dissolved oxygen (DO, ppm), electrical conductivity (EC, µS/m), pH, and water temperature (WT, Celsius) are monitored by the IoT sensor in real-time during the growing period. Repeated measure analysis is also performed to evaluate the plant growth performance. The result indicates that plant growth is attributed significantly to pH and EC values. A real-time data visualization and sharing platform is another avenue for the space farming ecosystem in the years to come.

## Graphical abstract

**Figure:**
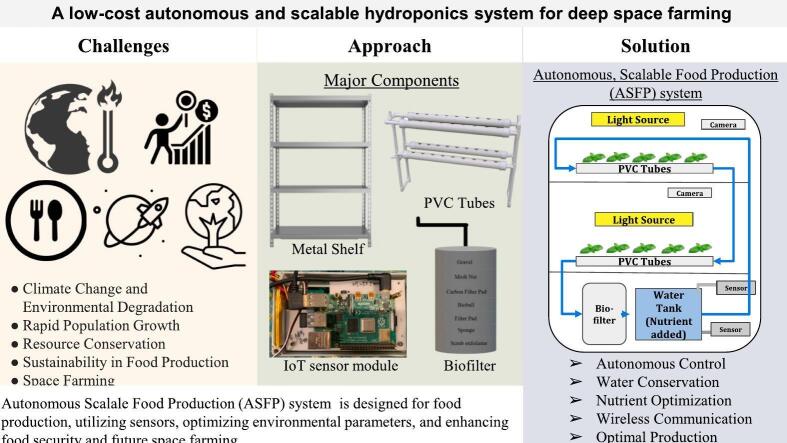

**Specifications table:** 

**Field**	**Description**
Hardware name	Autonomous, Modular, Scalable, And Soilless Food Production Platform (ASFP)
Subject area	• *Engineering and material science* *Environmental, planetary and agricultural sciences* *Educational tools and open source alternatives to existing infrastructure*
Hardware type	• *Measuring physical properties and in-lab sensors* *Other (please specify): Autonomous indoor farming system*
Closest commercial analog	No commercial analog is available.
Open source license	Free and open-source software licenses (mainly GPL).
Cost of hardware	$1121
Source file repository	https://doi.org/10.17632/frwfmmpy72.1

## 1 Hardware in context

Climate variability, population growth, and urbanization become key issues in agricultural sustainability. The decline in freshwater and soil nutrients is another challenge that the world is facing [1], [2]. Previous studies have indicated that the crop intensity has distinctly increased to meet the food demand target, consequently leading to soil exhaustion [3], [4] while the global population will reach 10.9 billion by 2050 [5]. Per capita consumption of food around the world continues to increase, resulting in the burden on crop production in farmlands [6], [7]. Extreme weather patterns induced by climate variability further drive food production challenges while increasing pest and disease risks is more present [1], [8]. The reduction in freshwater availability and decrease in agricultural yield will likely continue throughout the 21st century [9]. Under these unprecedented circumstances, alternative food production in a controlled environment is needed to produce reliable food in a changing global environment.

Since food security is critical in our modern life, the National Aeronautics and the Space Administration (NASA) initiated a centennial challenges program to seek innovations from diverse and non-traditional sources to explore revolutionary solutions to problems of interest to NASA and the nation [10]. As one of the programs, the Deep Space Food Challenge (DSFC) focuses on providing people in space and on Earth nutritious foods [11]. The research team from the University of Idaho in collaboration with the Rural Development Administration (RDA), South Korea participated in the Phase 1 and Phase 2 of the competition by proposing an autonomous, modular, scalable, and soilless space food production platform (ASFP) equipped with Internet of Things (IoT) sensors to grow basil in a controlled environment. The team was listed among the top 31 teams by DSFC and research activities are presented in this paper.

We selected Basil (*Ocimum basilcum*) for our experiment because it is a popular culinary herb in the mint family, commonly produced outdoors with a high demand for human consumption year-round [12]. Basil can be produced in controlled environments by providing suitable temperature and nutrients [13], [14]. To grow vegetables, there are several methods available, such as the nutrient film technique (NFT), deep flow technique (DFT), bag, and slab culture [15]. Among these, NFT and DFT are the most common methods for leafy crops like basil [13]. A study showed that hydroponic cultivation of the basil plants improved the antioxidant activity of aqueous and lipid extracts and increased the vitamin C, E, phenols, rosmarinic acid, and lipoic acid contents compared to the traditionally-grown basil in soil [16]. Thus, rosmarinic acid contents in the leaves, shoots, and roots of basil plants (*Ocimum basilicum L.*) were measured, and the highest rosmarinic acid content was found in the hydroponically-grown system during in-vitro multiplication [17].

Additionally, the advancement of smart controlled environment agriculture systems enables us to use real-time monitoring and optimization of environmental parameters [18]. The IoT sensors, in particular are helpful to monitor environmental parameters like pH, water temperature, electrical conductivity, and humidity for data acquisition and control [19], [20]. Cloud-based data visualization platform, such as ThingSpeak facilitate sharing of the data collected by IoT to any collaborators remotely. Open-source technologies in sensing and monitoring systems further justify enhancing automated hydroponic systems for urban farming through broader impacts. Since past research presents basil production in hydroponics systems reasonably well, we continued to use basil for the proposed scalable and autonomous food production system during this competition. This paper is organized as follows: 1) first, the system overview, including the components, modular design and technology advances, is described; 2) second, system constraints are addressed to grow vegetables with limited resources in terms of water supply, space concerns, and energy requirements followed by operational characteristics; 3) next, we demonstrate how basil can be grown in the proposed ASFP by meeting all necessary requirements and considerations set by NASA [11] finally, a summary and future work are discussed.

## 2 Hardware description

**Fig. 1:**
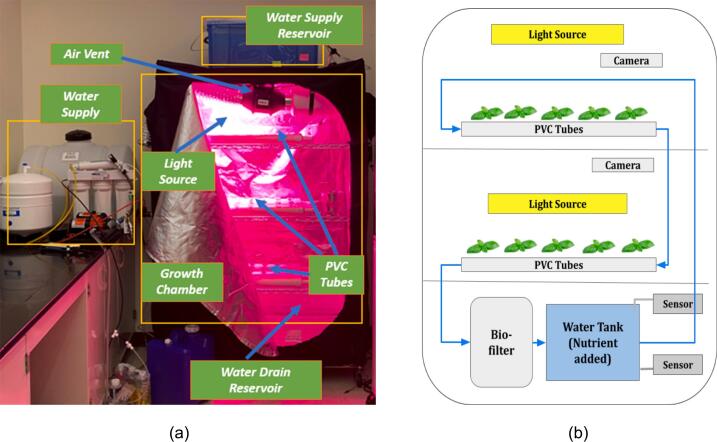
(a) Overview of the autonomous, modular, scalable, and soilless food production system (ASFP); (b) Schematic diagram of the plant growth chamber.

The ASFP is developed to grow leafy vegetables (basil) with low maintenance and automatic environmental control from seed to dining table for astronauts in space. The ASFP consists of three major components: the growth chamber, biofilter, and other hardware. The dimensions of the growth chamber is 123-centimeter width × 46-centimeter depth × 183-centimeter height, consisting of the National Sanitation Foundation (NSF)-certified steel wireframe. It is easily assembled with two crew members within 30 min. The weight of the growth chamber is about 28 kg (5.05 kg × 4 shelves + 0.70 kg × 8 supporting bars + 0.55 kg × 4 casters), but its weight can be reduced by using lightweight material (e.g., carbon fiber). Two layered Polyvinyl chloride (PVC) tubes and fittings are used to grow vegetables hydroponically with hardware accessories, including a water pump, timer, water tank (64-liter capacity), and supply hose lines (8.6-millimeter diameter) as shown in Fig. 1.

**Fig. 2:**
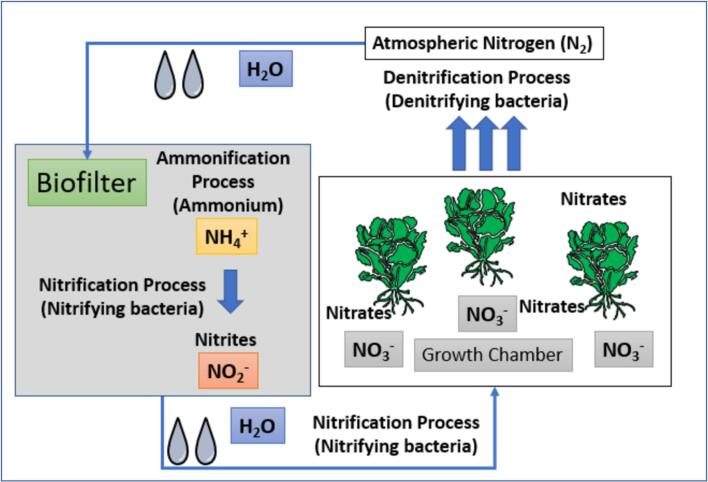
An example of nitrification–denitrification process in the controlled environment.

The main benefit of this ASFP is water conservation, as water is utilized for crop production in a closed recirculating system. Note that nearly no maintenance is required for this water recirculating system (See Fig. 2) besides replenishing the water supply about every 3–5 days depending on the water tank capacity. A nutrient mix in a solution containing nitrogen, phosphorus, and potassium with ratios 2–1-6 is used to maximize structural and foliar growth along with adequate light sources by meeting the power constraints (maximum draw of 3000 W and average draw <1500 W). A conventional light emitting diode (LED)-grow light was used to supply energy with optical lens technology and higher Photosynthetically Active Radiation (PAR) values for crop photosynthesis.

**Fig. 3:**
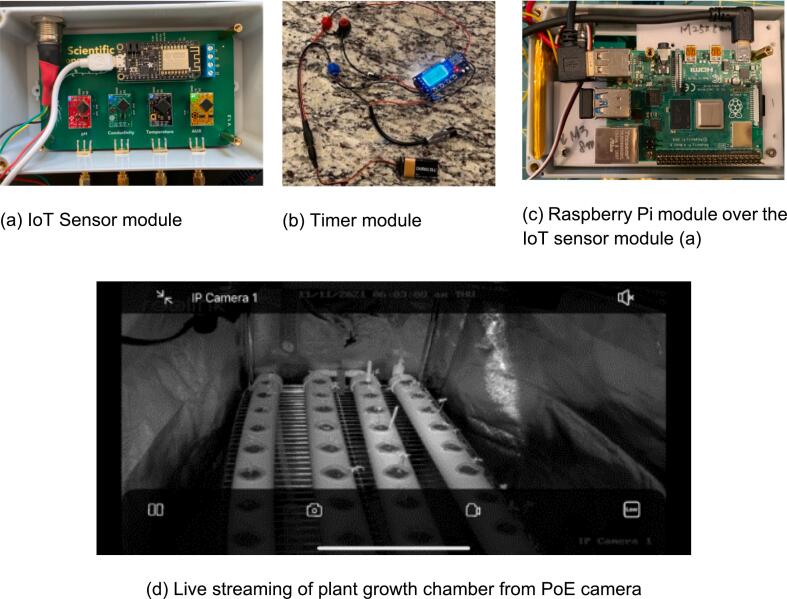
IoT Sensor setup and deployment in the ASFP.

For real-time monitoring and sensing, Internet of Things (IoT) devices are used to measure environmental data, including dissolved oxygen (DO), electronic conductivity (EC), pH, and water temperature (WT). Additionally, a Power over Ethernet (POE) Internet Protocol (IP) camera is also used to monitor growth stages in real-time and share these data with team members over the internet. Note that the built-in wireless communication in ASFP can also be useful for communicating between space crews and ground control centers on Earth via satellite uplink and downlink protocols. The Wi-Fi sensing board (SB), built-in Adafruit Huzzah32 (CPU), ESP32 communication module, and probes were used [21] to collect real-time water quality data, including DO, EC, pH, and WT. The ESP32, Raspberry Pi, and other open-source technologies offer low-cost, highly configurable, and accessible platforms that encourage innovation and collaboration. As shown in Fig. 3(a), the sensor ports, including pH, electrical conductivity, and auxiliary (AUX) ports are electrically isolated to ensure noise-free electrochemical readings while the Printed Circuit Board (PCB) is powered and programmed through a panel-mount USB connector with the Central Processing Unit (CPU) by supplying 5 V to the power bus. Necessary efforts were made to calibrate the probes based on the instructional manual before they came into contact with water. A Raspberry Pi 4 (RP) [22] was used to transmit the collected data from SB in Arduino [23] to the cloud-based data sharing platform (e.g., Thingspeak, Amazon AWS) over the internet network (See Fig. 3).

## 3 Design files summary

The suggested ASFP can be constructed without the need for 3D-printed components or design files. All necessary parts are readily available for purchase at local appliance and hardware stores or through online marketplaces.

## 4 Bill of materials summary

Table 1 details the full bill of materials required to build the ASFP. The components specified are readily available and can be sourced from a variety of local and online retailers, including Amazon.

**Table 1:** Bill of materials summary to build the ASFP.

Designator	Component	Number	Cost per unit currency (US$)	Total cost currency (US$)	Source of materials	Material Type
Seed Germination Kit	Heat Mat, Germination Tray, Plastic Dome, Fluorescent Light	1	$55.90	$55.90	Online Store (https://a.co/d/gMEId9J)	Polymer, Plastic
Grow Plug	Rockwool	2	$9.99	$19.98	Online Store (https://a.co/d/2xpIsiy)	Mineral-based material
Basil Seeds	−	1	$4.99	$4.99	Online Store (https://a.co/d/7jSl6Wb)	Seeds
Alloy-Steel Shelf	Zinc Coated Steel Shelf (4 tiers)	1	$139.98	$139.98	Online Store (https://a.co/d/dfOPFOd)	Metal (Steel, Zinc Coated)
PVC Pipes (6 pipes)	PVC	1	$54.99	$54.99	Online Store (https://a.co/d/1qHDleq)	Polymer (PVC)
Biofilter	Scrub exfoliator (6 in bundle)	2	$9.99	$19.98	Online Store (https://a.co/d/dTYelDt)	Polymer
	Sponge	5	$11.95	$59.75	Online Store (https://a.co/d/iT8KzWZ)	Polymer
	Bioball	1	$89.92	$89.92	Online Store (https://a.co/d/5A1AqVP)	Polymer
	Filter Pad	1	$28.11	$28.11	Online Store (https://a.co/d/gKcKL7f)	Polymer
	Bucket	1	$4.48	$4.48	Online Store (https://www.homedepot.com/p/The-Home-Depot-5-Gallon-Orange-Homer-Bucket-05GLHD2/100087613)	Polymer (Plastic)
LED Grow Light	−	1	$128.99	$128.99	Online Store (https://a.co/d/8u9n9gt)	Electrical Component
IoT Sensor for Water Quality	Sensor kit	1	$499.99	$499.99	Online Store (https://bityl.co/AkXJ)	Electrical Component
Nutrient Solution	−	1	$13.99	$13.99	Online Store (https://a.co/d/agUEnNC)	Chemical Solution

## 5 Build instructions

To assemble a multiple-layer steel shelf, start by laying out all the parts to ensure everything is included. Begin with the bottom shelf by attaching it to two vertical supporting legs using snap-in fittings, leaving them slightly loose for adjustments. Next, add the other two supporting legs at the remaining corners of the shelf, securing them similarly. This establishes a stable base for the rest of the structure. Install the remaining shelves by determining desired heights and attaching each shelf to the support layers using snap-in fittings. It's important to ensure that each shelf is level before fully engaged between layers and supporting legs. Use a level tool to verify this. Once all shelves are in place and aligned correctly, go back and make sure all other snap-in fittings are in place to secure the structure. Finally, stand the shelf upright, check its stability, and make any necessary adjustments to keep it level. Once the shelves are complete, two-layered PVC tubes and fittings are positioned on layers with hardware accessories, including water pumps, time, water tank, and hoses to grow vegetables. The water pump was installed inside the water tank, hoses are attached to the pump and routed through the PVC tubes for irrigation, and the timer is configured to automate watering cycles (5 min of water supply every 30 min). The biofilter is another critical component for recycling water within the ASFP system. A biofilter composed of multiple layers of fabric material is then inserted into the shelf to complete ASFP building. The multiple layers of fabric material, such as a filter pad, scrub exfoliator, and bioball, are used and set up in a utility bucket (22.7 L, equivalently 5 gallons) to facilitate nitrification cycles by converting wastewater to treated water for the growth chamber. The building procedure of the ASFP is available at https://www.youtube.com/watch?v=XpzVLWrcKVo.

## 6 Operation instructions

Minimal effort is needed to operate ASFP from seedling to the dining table. The initial setup begins with adding seeds in the heat mat for propagation in a 508-millimeter length × 245-millimeter width × 177-millimeter height plastic dome and T5 fluorescent grow light (17-watt energy consumption). This takes less than 10 min for one person to complete. Once the initial setup is complete, vegetable seeds (2–3 seeds) are inserted into each growing media plug (GMP) in a 25-millimeter width × 25-millimeter length × 25-millimeter height hand-made with rockwool coconut fiber (RCF). Adequate amounts of tap water are then applied to moisturize the seed for germination. Note that this whole setup takes less than an hour when worked by one person. About 2 L of water are needed for every 150 seeds in 50 GMP cubes as it takes 9–10 days from seedling to transplanting via germination. Every morning, additional watering (about 250 ml) is needed, but automation in the water supply is achievable through a timer-engaged, small water pump (e.g., 220 L per hour with 12–24 DC power) to minimize efforts when needed. Once transplanting is complete after 9–10 days, plants will grow in the growth chamber as part of a recirculating closed system. Other than water and nutrient solutions (500 ml solution per 64-liter water tank), there are no other fluids or inoculation process required. System requirements and constraints are described briefly below.

• Volume: The dimensions of the proposed ASFP system are 123-centimeter width × 46-centimeter depth × 183-centimeter height so that it can pass through the spacecraft doorway constraint (107-centimeter width × 190-centimeter height). The volume of the ASFP per unit is about 1 cubic meter.
• Power: Maximum draw of power is set to 3000 W, while the average draw should be less than 1500 W
• Water: Net consumption of water is not constrained, but it is measured by the following equation: C_net = initial water input + “new water” added over time.
• Mass: Total mass of the single ASFP unit is about 47.6 kg and other elements include: 1) Growth Chamber: 28 kg, 2) PVC fittings: 10 kg, 3) Seedling heat mat and GMP: 3 kg, 4) Water tank: 1.5 kg, 5) Light source: 2.5 kg, 6) Water pump, biofilter, and 7) IoT sensors (optional): 2 kg.
• Data Connection: Operational data are collected using IoT devices, including water quality probes and a live-feed video camera, to wirelessly transmit data to a remote location so that collaborators can share the data over the internet (e.g., Thingspeak, Amazon Web Services).
• Crew Time: The seedling and germination stage requires an average of 10 min per person to supply water. Once transplanting is complete from the seedling pad to the growth chamber, nearly no crew time is needed except adding additional water (e.g., 30-liter around Day 15) because the proposed ASFP will be operated autonomously.
• Other operational constraints: The proposed ASFP system is running in a climate-controlled room at 22 degrees Celsius and 50 percent relative humidity as operational constraints. These two environmental parameters (temperature and relative humidity) are also measured by IoT sensors in real-time wirelessly and it is shared with collaborators remotely. We assume that the proposed ASFP system will be operational in a space-like environment near future, although it is currently based on Earth’s gravity (9.81 m/s^2^) and ambient atmospheric conditions of 101,325 Pascals.

In contrast to conventional farming practices, the proposed ASFP is an autonomous, modular, scalable, transportable, sustainable, and highly efficient system to be operable in space. Once transplanting is complete after germination, food production can take place in full automation. Thus, necessary nutrients (N-P-K), water supply, and light sources for photosynthesis are controlled by IoT sensor devices. Additionally, the ASFP is compatible with the Digital Twin platform [24], [25], [26], which can be updated and controlled remotely by the ground control station on Earth (when needed). Thus, different perspectives and various comments from plant scientists on earth can also be incorporated into the operational stage in space.

## 7 Validation and characterization

### 7.1 Temperature and humidity

There was no significant water and energy consumption observed in the system because ASFP is recirculating water using a small water pump (e.g., 220-liter per hour). Average values of temperatures and relative humidity in the environmentally-controlled room is maintained range between 20–24 °C and 48–62 %, respectively, for the growing period. No significant spatial temperature and relative humidity differences within the growth chamber were observed.

### 7.2 Water and power consumption

About 2 L of water are needed for 150 seeds in 50 GMP during the seedling stage, while an additional 64 L of fresh water were added to the reservoir to recirculate water for reuse. Water recycled within the ASFP unit is not considered “new water,” so total water consumption is estimated to be about 80 L from seedling to harvest for 50 basil sites (0.6 L per basil). Thus, about 40 % of water was consumed by plants through their evapotranspiration activities. Power consumption was also observed to estimate energy footprints. LED-grow lights composed of 200 pieces of 15-watt LED light bulbs are used to maximize basil growth. The average power consumption for fast gemination draws a maximum of 16 W, while approximately 220 W are required for plant biomass during the growth stage in ASFP. Overall, the proposed energy footprint adequately met the power constraint described above. An on/off time switch was used to control the LED grow lights for 12-hour intervals (6:00 AM–6:00 PM Power ON; 6:00 PM–6:00 AM Power Off).

### 7.3 Nutrient supply

A nutrient mix in a solution containing nitrogen (N), phosphorous (P), and potassium (K) with ratios 2-1-6 is used to maximize structural and foliar growth [27]. To ensure the best results, adequate water needs to be added to a reservoir and mixed well before applying. Based on directions, only 300 ml was used to grow 50 GMP for this study.

### 7.4 Plant growth process

Basil growth in ASFP requires little attention as opposed to some filed crops for extended periods of time. For a healthy seedling stage, temperature, moisture, and light sources are critical for the germination period. Thus, basil seeds should be in place in some medium, such as germination plugs or soilless potting box, covered by a wet towel and plastic humidity dome for the first 2–3 days. Once seeds start to germinate, the wet towel should be removed to allow space for basil to grow. Although substantial amounts of water are not required during the germination period, adequate moisture levels should be maintained by adding water regularly (e.g., once a day). Depending on the application, water can be supplied gently by hand, with an overhead mister, sub-irrigation, or drip irrigation method when applicable. Tap water or bottled drinking water would suffice and no additional nutrient solution is needed at this stage; the salts in the nutrients may prevent the seeds from taking up water, ultimately resulting in less vigorous root growth. A heat mat is helpful for facilitating uniform germination processes. Once germination is complete, supplemental light sources (e.g., T5 fluorescent tube light) are recommended while the wet towel is no longer needed.

About 10 days after seedling, basils were transplanted to ASFP. At this stage, the roots are visible on the outside of the GMP, and true leaves are flourishing (Not shown in this paper). To achieve a successful, stress-free transplant, moving between growing mediums (e.g., GMP) should be avoided when possible. Thus, simply break individual RCFs by hand and places them on GMPs.

As soon as the transplant is complete, key environmental variables are monitored. Thus, in addition to indoor climate (e.g., air temperature and humidity) in the ASFP, water quality components, including DO, EC, pH, and WT are measured in real-time using IoT sensors [21]. Previous studies reported that basil plants are suitable for indoor gardening within various environmental conditions ranging from 10 to 27 °C for air temperature, EC from 0.5 to 4.0 dS.m^−1^, pH from 4.3 to 8.2, and daily light integral (DLIs) as low as 4 mol.m^−2^ d^−1^ [13], [28], [29], [30], [31].

It might be useful to quantify light levels in units referred to as the DLI for energy footprints. DLI is the amount of photosynthetically active radiation (photons), which are individual particles of light in the 400–700 nm range that plants receive over 24 h [32]. It is typically computed by converting Photosynthetic Photon Flux Density (PPFD) using the equation [33]:

(1) DLI (mol/(m^2^.day) = 3.6 × 10^–3^ PPFD (micromole/(m^2^.s)) × Light-hours/day

where, 3.6 × 10^–3^ is a converting factor associated with mole and time (e.g., from hours to seconds).

A light meter was used to measure DLI as moles of light (mol) per square meter (m2) per day, and an average DLI of 18 was recorded. Note that plants in green-houses typically experience about a 40 % reduction in DLI below that of outdoor levels due to glazing and shading from the structure [34]. Light color is another factor to consider when growing basil; leafy greens fare best with lights emitting a higher proportion at the blue end of the spectrum (450–496 nm), which facilitates vegetative growth. During the whole growth period, a light spectrum with blue/red LED was used and a day length of 12 h. Note that no separate experiment was conducted to compare basil growth associated with a different light source.

### 7.5 Statistical analysis of growth parameters

#### 7.5.1 pH

**Fig. 4:**
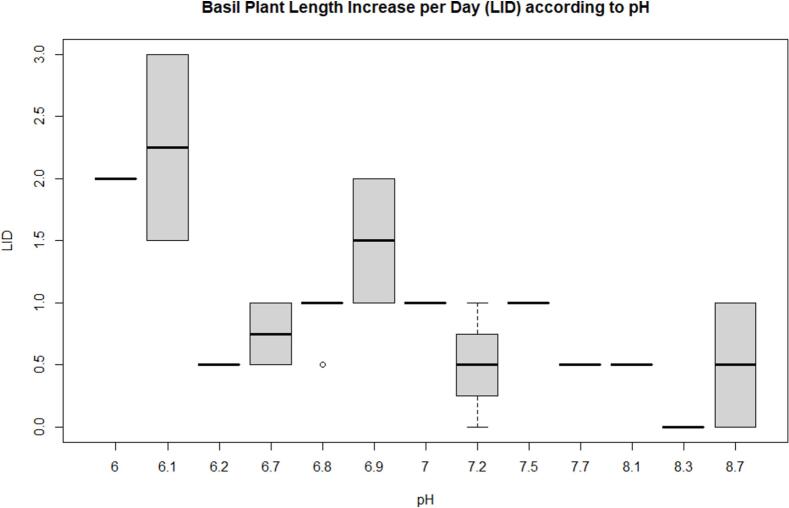
Boxplot between pH and Plant Length Increase per Day (LID, centimeter).

Nutrient solution application in ASFP is found to change the pH values that impact plant growth. The pH of the system varies between 6 (lower limit) to 8.7 (upper limit), but it appears that the performance of the basil plant is greatest with pH 6.1 as shown in Fig. 4. Basil has been found to grow optimally at a pH range of 5.5 to 6.5 in previous studies [35]; therefore, the nutrient solution maintaining a pH of 6.1 contributed to the increased basil plant length in our study.

Table 2 shows the ANOVA table with a fixed effect model using LID and pH values, which is a significant result (F = 30.65 when df = 1 and 18) with 99 % confidence level.

**Table 2:** ANOVA table (Type 3 tests) for LID as a function of pH.

Effect	df	MSE	F	ges	p.value
LID	5, 18	0.83	2.88*	0.443	0.044
pH Sensors	1, 18	0	30.65***	0.01	<0.001
LID:pH Sensors	5, 18	0	0.9	0.002	0.499

Signif. codes: 0 ‘***’ 0.001 ‘**’ 0.01 ‘*’ 0.05 ‘+’ 0.1 ‘’ 1.

Sphericity correction method: Greenhouse-Geisser.

**Fig. 5:**
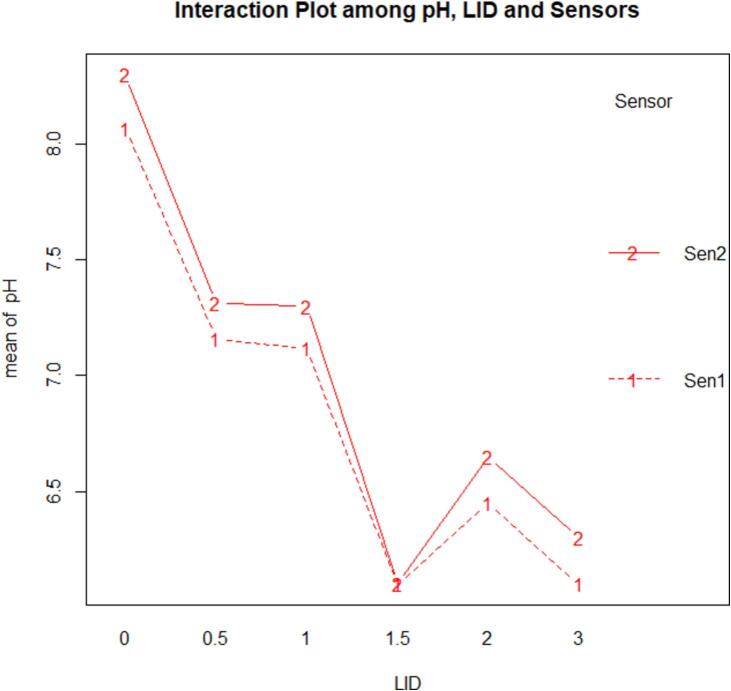
Interaction plot between pH and LID.

The interaction plot is shown in Fig. 5. Also shows a significant difference in LID (centimeter) as pH decreases, recorded by both sensors. Therefore, a pH value less than 6.1 would be a good environment for a higher yield of basil growth.

#### 7.5.2 Electrical conductivity

**Fig. 6:**
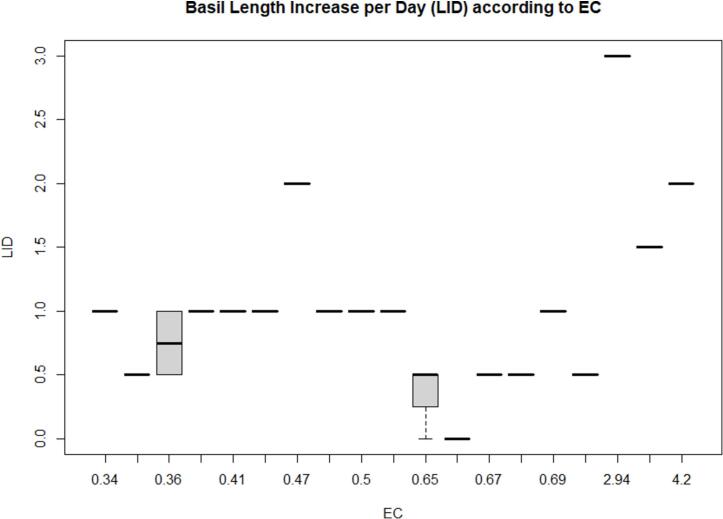
Boxplot between EC (S/cm) and Plant Length Increase per Day (LID, centimeter).

It is noted that basil plant growth is also sensitive to EC values driven by nutrient concentrations. Thus, the higher yield was observed as EC values (e.g., 2.9 above) increased as shown in Fig. 6. Previous studies have demonstrated that basil can achieve optimal growth and yield with high electrical conductivity (EC) levels in hydroponic systems. For example, Ren et al. [36] showed that an EC of 3.0 dS·m^−1^ significantly promoted basil yield than the lower EC levels (0.5 and 1.0 dS·m^−1^). Also, Morano et al. [37] stated that the highest basil yield, both in whole plants and leaves, was achieved at EC of 2.8 mS·cm^−1^ compared to the lower ones. Therefore, our findings shown in Fig. 6 are aligned with the previous studies and suggest that the growth of basil plants can be benefitted from moderately high EC levels.

Table 3 shows the ANOVA table from the fixed effect model using LID and EC values, indicating significant effects (p-value < 0.01).

**Table 3:** ANOVA table (Type 3 tests) for LID as a function of EC.

Effect	df	MSE	F	ges	p.value
LID	5, 18	0.63	4.88**	0.534	0.005
EC Sensors	1, 18	0.12	60.11***	0.341	<0.001
LID:EC Sensors	5, 18	0.12	11.27***	0.327	<0.001

Signif. codes: 0 ‘***’ 0.001 ‘**’ 0.01 ‘*’ 0.05 ‘+’ 0.1 ‘’ 1.

Sphericity correction method: Greenhouse-Geisser.

The other two controlled factors (DO and WT), however, don’t necessarily show significant results. In general, basil grows well uniformly in water temperature range from range 22 °C to 27 °C. But the result shows that there is no statistical significance between LID and water temperature (Not shown in this paper). A similar result was found in DO in the sense that plan yield doesn’t respond significantly to DO values. Perhaps, this is relevant to photosynthesis which requires carbon dioxide rather than oxygen.

### 7.6 Food safety

For food safety, we performed additional lab testing for potential contaminations with harmful microorganisms, including salmonella spp., Escherichia coli O157:H7, listeria monocytogenes, and bacteria (e.g., enterobacteriacese). Lab testing reports provided by a certified lab accredited to the International Organization for Standardization (ISO)/International Electrotechnical Commission (IEC) standard 17025:2017(E) indicated that no harmful microorganism was found in the submitted samples (Not shown in this paper).

### 7.7 Data visualization via cloud-based data sharing

**Fig. 7:**
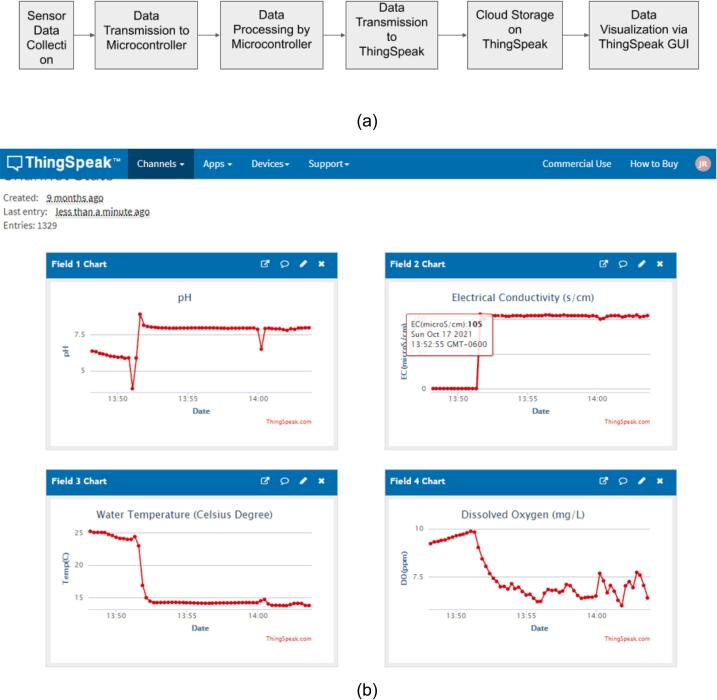
(a) Flow Chart of data visualization and sharing process. (b) An example of ThingSpeak’s Graphical User Interface shows water quality data (DO, EC, pH, and WT).

ThingSpeak [38] is used to send water quality data, DO (ppm), EC (µS/m), pH, and WT (Celsius) to cloud networks for visualization and data sharing with collaborators around the world or in between Earth and space. It appears that average values for water quality components are about 6.5, 385, 6.2, and 17 for DO, EC, pH, and WT, respectively, which are shared with collaborators via ThingSpeak. ThingSpeak is an open-source IoT analytics platform service that allows aggregate, and visualize real-time data streams in the cloud [38]. Basically, all water quality data measured by IoT probes (Arduino, 2021) are transferred to the flexible ThingSpeak cloud services over the internet. Fig. 7(a) shows the flow chart that depicts the steps conducted by the sensors and the ThingSpeak platform for data monitoring and sharing while Fig. 7(b) shows a typical example of ThingSpeak’s graphical user interface (GUI) from a web browser’s interface (Web). Note that all water components, including DO, EC (µS/m), PH, and WT (Celsius) are displayed nicely (See Fig. 7(b)). As default, these data are updated every 15 s, but time-step can be adjustable in user settings.

## 8 Conclusions

Climate variability and land use change continue to threaten food security around the world. Due to limited natural resources (e.g., water and land), sustainable food production becomes more difficult to meet demand targets driven by increases in population. To mitigate such impact, an alternative food production system in a controlled environment might be a promising solution to grow vegetables efficiently without much water and energy inputs. In line with this critical issue that we face today, NASA initiated a food production challenge in space. As a team from the University of Idaho in collaboration with the Rural Development Administration, we developed an autonomous, modular, scalable, and soilless space food production platform (ASFP) equipped with IoT sensors to grow basils. Overall, the proposed ASFP using hydroponics worked very well by meeting the system requirements and constraints set by NASA, including doorway constraint, energy footprint, limited water consumption, total mass, and crew time. Ambient indoor environmental conditions for temperature and humidity have been maintained at a range from 20–24 °C and 48–62 %, respectively, for the growing period. Water quality data, including DO, EC, pH, and WT are monitored using IoT sensors in real-time and then sent to the ThingSpeak cloud for collaborative sharing around the world or between Earth and space. Average values of these data are recorded at 6.5, 385, 6.2, and 17 for DO, EC, pH, and WT, respectively. Based on statistical analysis, it appears that basil yield responds to pH and EC values significantly while DO and WT is less sensitive for basil growth.

The proposed ASFP can be also used to grow various crops (e.g., potato, tomato, other leafy vegetables) for commercial production [39]. However, to achieve fully autonomous food production systems in space, additional sensors, such as an optical sensor, light control sensor, and/or vision sensor, may be useful to better characterize optimal growth stages. In future studies, our research will explore the effects of microgravity on system performance to expand its applicability in space agriculture. Additionally, food production equipped with a digital twin (DT) platform would be a useful exercise to improve the ASFP by implementing the next generation of data, models, and decision support tools for future agricultural production systems. The DT is an excellent platform to make alterations and adjustments that would be too expensive or risky for real, physical objects [33], [34], [40], [41], [42], [43], [44], [45], [46]. Applications of DT in the proposed ASFP would be another avenue to grow crops on earth and space in a sustainable manner near future. To improve visualization and simulation for larger projects within DT platforms, enterprise-scale web services, such as Amazon Web Services (AWS) or Microsoft Azure web apps, could be considered to ensure deliverables seamlessly online. Finally, the authors anticipate that the proposed ASFP will contribute to indoor farming communities (e.g., hydroponics and/or aquaponics with fish and vegetables) through crop growth optimization by minimizing costs.

## 9 Ethics statements

No ethical approval is needed.

## 10 Funding support

This work was carried out with the support of “Cooperative Research Program for Agriculture Science and Technology Development (Project No. PJ015553)”, 10.13039/501100003627Rural Development Administration (RDA), Republic of Korea. J.H.R. is also supported partially by the National of Food and Agriculture, 10.13039/100000199United States Department of Agriculture (USDA), under award 2023-69018-41016 (system conceptualization and design) and under ID01654 (data acquisition and analysis). Any opinions, findings, conclusions, or recommendations expressed in this publication are those of the authors and do not necessarily reflect the views of RDA or USDA.

## CRediT authorship contribution statement

**Jae Hyeon Ryu:** Investigation. **Jeonghyun Baek:** Resources. **Zarin Subah:** Data curation.

## Declaration of competing interest

The authors declare that they have no known competing financial interests or personal relationships that could have appeared to influence the work reported in this paper.
